# Acoustic accessibility and staff knowledge on hearing and communication in long-term care facilities: evaluation of a preventive training program

**DOI:** 10.1038/s41598-026-60991-z

**Published:** 2026-07-16

**Authors:** Carolin Gravel, Elena Pützer, Karolin Schäfer

**Affiliations:** 1 Institute for Special Needs Education, D/deaf and Hard of Hearing, University of Duisburg-Essen, Berliner Platz 6-8, 45127 Essen, Germany; 2 Faculty of Human Sciences, University of Cologne, Cologne, Germany

**Keywords:** Age-related hearing loss, Long-term care, Staff training, Acoustic accessibility, Health care, Health humanities

## Abstract

Age-related hearing loss is highly prevalent among older adults. Long-term care (LTC) residents are an especially vulnerable population, yet hearing accessibility and staff expertise in hearing care often remain insufficient. This study examines (1) the structural and acoustic accessibility conditions in LTC facilities, (2) the hearing-related knowledge and training needs of staff, and (3) staff assessment of feasibility and sustainability of a preventive training program. A mixed-methods study was conducted in 74 LTC facilities in Germany. Data were collected pre- and post-intervention and at one year’s follow-up. Data sources included questionnaires, quantitative surveys, room acoustic measurements, and qualitative interviews. Quantitative data were analyzed descriptively; qualitative data were interpreted via structuring qualitative content analysis. Most facilities (81%) lacked acoustic accessibility concepts. More than half of the staff had limited knowledge on hearing and communication and expressed high training needs on this topic prior to the training program. Post-intervention interviews highlighted organizational barriers, but also a strong increase of awareness. Follow-up findings indicated moderate improvements in hearing-related practices and communication after the training program, though structural acoustic changes remained difficult to implement sustainably. The results show that improving hearing accessibility in LTC requires an integrated approach that strengthens both acoustical conditions in the facilities and staff competencies.

## Introduction

The risk of hearing loss increases with age, and studies show that age-related hearing loss is highly prevalent among older adults^1–4^. Age-related hearing loss results in the greatest societal and economic burden of hearing loss across the life span because of the high prevalence. Demographic shifts are expected to compound these problems^5^, a person-centred ear and hearing care is needed, especially in higher ages. The WHO states that over 58% of all moderate or higher grades of hearing loss worldwide occur in adults over the age of 60. In the group of people between 85–89 years, over half of the individuals (51.8%) are affected by moderate or higher grades of hearing loss^5^. Research indicates that the provision of hearing devices, particularly for elderly adults, is inadequate, for example, due to inaccessible hearing healthcare services, personal reservations, lower technology commitment, or socioeconomic status^2,6,7^.

The high prevalence of hearing loss in older adults poses an important need for action for the care sector. According to the Federal Statistical Office, 5.7 million people in Germany had care needs at the end of 2023. Of these, 14% lived in a long-term care (LTC) facility. More than half (53%) of the residents in long-term care facilities were elderly people, aged 85 and older^8^. Many residents in LTC facilities experience some grade of hearing loss – whether diagnosed or undetected and/or untreated^9–11^. Studies show that hearing loss can severely affect communication, participation, well-being, and cognitive functioning^12–18^. These effects of hearing loss can significantly impair caregiving routines in LTC facilities and thus reduce the quality of life of the residents. National and international research emphasizes the need for staff training, well-structured hearing management, and accessible acoustic environments in LTC facilities^9,10,16,19–34^.

However, few large-scale evaluations have assessed these dimensions comprehensively in this specific setting. Based on this knowledge, the “Blindeninstitut Würzburg” (Institute for the Blind in Würzburg) developed the preventive training program “Hearing and Communication in Long-Term Care Facilities” (*Hören und Kommunikation in Pflegeeinrichtungen*) in cooperation with a research team of the University of Cologne and later Duisburg-Essen. The components of the preventive training program were.

1. An organizational analysis of the situation in LTC facilities regarding hearing and communication,
2. Staff training on hearing health, communication with people with hearing loss, and hearing devices,
3. Assessment of room acoustics and acoustic accessibility,
4. Assessment of ear and hearing health care of the residents.

The goal of the preventive training program was to raise awareness about hearing loss and its effects, strengthen staff expertise, assess acoustic accessibility, and improve documentation routines across facilities. Moreover, several residents had the chance to have their hearing tested by an acoustician. A multi-professional team of the Blindeninstitut Würzburg facilitated the two-day preventive training program with all components at the LTC facilities and distributed the evaluation questionnaires. A qualitative interview study and a follow-up online survey were carried out by the research team.

This paper presents a focused analysis on three central domains:

- The staff’s expertise and training needs on topics of hearing and communication,
- The facilities’ acoustic and environmental accessibility and
- The staff’s experiences with the implementation of the programs’ recommendations.

## Methods

### Study design

**Fig. 1:**
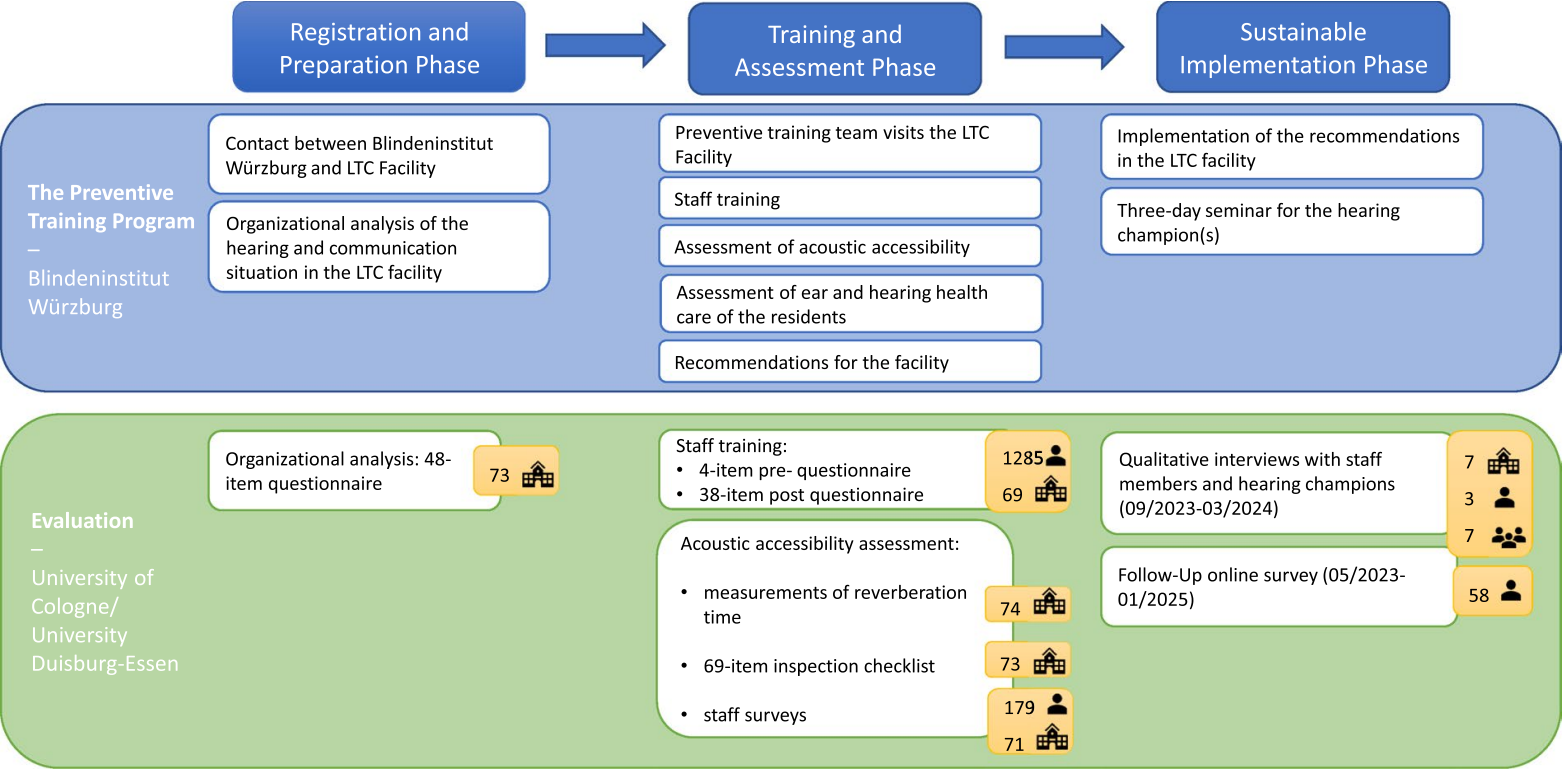
Study flowchart.

The present study used a mixed-methods evaluation, integrating quantitative surveys, room acoustic measurements, and qualitative interviews (Fig. 1). The evaluation was carried out in parallel with the preventive training program to identify gaps and to make changes along the way if necessary (formative evaluation). At the end of the project, a final evaluation of all components was conducted to report on impacts of the preventive training program (summative evaluation).

### Setting and participants

The study included *n* = 74 LTC facilities in Bavaria visited by the team of the Blindeninstitut Würzburg between October 2021 and September 2024 as part of the preventive training program “Hearing and Communication in Long-Term Care Facilities”. Information about the preventive training program was distributed to long-term care facilities throughout Bavaria. The sample was collected through convenience sampling. Ultimately, a wide range of small and large facilities in both urban and rural areas participated. The costs were covered by five long-term care insurance providers (AOK Bayern, Betriebskrankenkassen in Bayern, IKK classic, KNAPPSCHAFT, and Sozialversicherung für Landwirtschaft, Forsten und Gartenbau). The LTC facilities chose participating staff members and hearing champions based on availability.

Staff participants included nurses, social care assistants, facility managers, and quality management personnel. There were no exclusion criteria for the staff participants. Every facility had the opportunity to choose at least one of their staff members as a “hearing champion” who received extended training in a three-day seminar on hearing and communication in older age at the Blindeninstitut Würzburg.

### Data collection

Data was collected through surveys with the LTC facility staff, an acoustic accessibility assessment, qualitative interviews, and an online survey. During the implementation of the preventive training program and the evaluation, COVID-19 restrictions limited data collection at some facilities. Moreover, some facilities were understaffed while the preventive training program took place. Because of these issues, the project team had to work with varying sample sizes, as some facilities did not participate in all parts of the program.

#### Organizational analysis

To analyze the organizational structure of the LTC facilities, the project team developed a 48-item questionnaire with mostly dichotomous yes-or-no scales and space for follow-up information, e.g., which kind of software the facility is using for their documentation. It assessed documentation routines (21 items); available support structures, e.g., existing collaboration with Ear, Nose, and Throat (ENT) specialists and acousticians (9 items);existing room acoustics concepts (4 items); number of employees and staff knowledge about hearing and communication (6 items); as well as resident structure (number and care level) and communication formats used in the participating facilities (8 items). The questionnaire was sent to the LTC facility in the preparation phase of the program.

#### Staff training surveys

The project team developed a 4-item pre- and a 38-item post-questionnaire for the staff training. In total, *n* = 1285 staff members in *n* = 69 facilities participated in the in-house training. The pre-training questionnaires evaluated staff knowledge about hearing and communication, training needs, and interest in training on a 1 to 10 scale, as well as the individual expectations towards the training (open-ended question) prior to the program. Post-training questionnaires assessed demographic data (6 items), perceived relevance of the training in relation to their everyday work (8 items), satisfaction with the training regarding the content and the presentation (7 items), the quality of information (5 items), and the practical relevance (5 items). All items were rated on a 4-point Likert scale from “I completely agree” to “I completely disagree” and an alternative option, “I cannot make a statement on this”.

Moreover, there were 6 open-ended questions and a brief check on the reasons for not filling out the questionnaire completely or at all. The participating staff members filled out the post-training questionnaire right after the training.

#### Acoustic accessibility assessment

Acoustic accessibility of the LTC facilities was assessed through measurements of reverberation time and an inspection checklist. Using a sound level meter, the preventive program team determined the reverberation time in several frequently used rooms within the LTC facilities. In order to facilitate the measurement, the rooms had to be unoccupied. The data was then processed in a diagram via special software and compared with the values required for public buildings by the German DIN standard 18041 (‘Acoustic quality in rooms’). The requirements are categorized depending on the size and type of use of the rooms, e.g., a spacious dining hall or a small meeting room. We compared the measured values with the requirements for rooms for so-called ‘inclusive communication’ (usage type A4 of the DIN standard)^35,36^. ‘Inclusive communication’ takes particular account of the needs of people with hearing loss or hearing difficulties. The team conducted measurements in *n* = 277 rooms across 74 LTC facilities.

The project team developed a 69-item checklist with a dichotomous yes-or-no scale to evaluate accessibility features of the facility such as visual signals, sound-absorbing elements, and background noise (*n* = 73). In addition, participating staff received self-administered staff surveys before and immediately after the acoustic accessibility assessment (*n* = 179) in 71 LTC facilities. The pre-assessment questionnaire (4 items) evaluated staff knowledge about accessible room acoustic concepts, the perceived necessity of and interest in an acoustic accessibility assessment on a 1 to 10 scale, as well as the individual expectations towards the assessment (open-ended question). The post-assessment questionnaire included items regarding demographic data (6 items) and ratings of the assessment regarding the content and recommendations of the assessment (8 items). The items were scored on a 4-point Likert scale (“I completely agree”, “I tend to agree”, “I tend to disagree”, “I completely disagree”) with an alternative option, “I cannot make a statement on this”. Moreover, there were 6 open-ended questions regarding the individual experience of the assessment.

#### Qualitative interviews

To identify sustainable changes and challenges in implementation of the recommendations of the preventive training program, the project team planned a qualitative interview study within at least 5 LTC facilities. 13 facilities were invited to participate. between May and October 2023. To ensure comprehensive results, the facilities were selected based on the following criteria: size of facility, number of residents, and location (urban/rural). Eventually, 7 LTC facilities agreed to participate. The preventive training program took place in these facilities 8–16 months prior to the interviews. The research team (author CG and a student research assistant) conducted seven problem-centred group interviews with *n* = 24 staff members as well as *n* = 3 individual interviews with “hearing champions” between September 2023 and March 2024. The participants were selected by the invited LTC facilities. 2 of the group interviews were run via the video-chat platform Zoom, whereas the other interviews were conducted face-to-face in the LTC facilities.

#### Follow-up online survey

From May 2023 until January 2025, the research team ran an anonymized online follow-up survey and invited contact persons from all LTC facilities to participate in the survey. An invitation was sent to 162 persons; 39 times email delivery was not successful. Eventually, *n* = 58 staff members participated in the follow-up online survey. This corresponds to a response rate of 47%. The online survey included questions about long-term changes in hearing care practices, cooperation networks, and modifications regarding acoustic accessibility. Figure 1 provides an overview of the programs’ structure.

### Data analysis

Quantitative data were analyzed descriptively using Microsoft Excel. Qualitative interview data were transcribed and analyzed via structuring qualitative content analysis following Kuckartz and Rädiker^37^.

### Ethical consideration

This study, with all its components, written informed consent, questionnaires, and interview guide, was approved by the Ethics Committee of the Faculty of Human Science of the University of Cologne (approval number SJHF0137). All methods were performed in accordance with the relevant guidelines and regulations. Furthermore, all local legal and regulatory requirements were fulfilled. Informed consent was obtained prior to the study from all participants and/or their legal guardian(s).

## Results

### Staff’s pre- and post-training hearing-related expertise and training needs

In the organizational analysis, the majority of LTC facilities (93%) stated that staff members were informed about hearing devices of residents, but in 60% of the LTC facilities, the employees were not specifically trained regarding age-related hearing loss, effects of hearing loss, and communication strategies. 93% of the LTC facilities declared that staff were unfamiliar with social rights or support systems available for residents with hearing loss. Data from the staff training surveys revealed that most of the participants of the in-house staff training were female (76%; male: 15%, diverse: 0.2%). Of *n* = 1181 staff members with available information on age, the mean age was 44 years (min. 16 years, max. 78 years). On average, about 19 employees per facility participated in the staff training program (min. 1; max. 52).

#### Pre-training staff survey

**Fig. 2:**
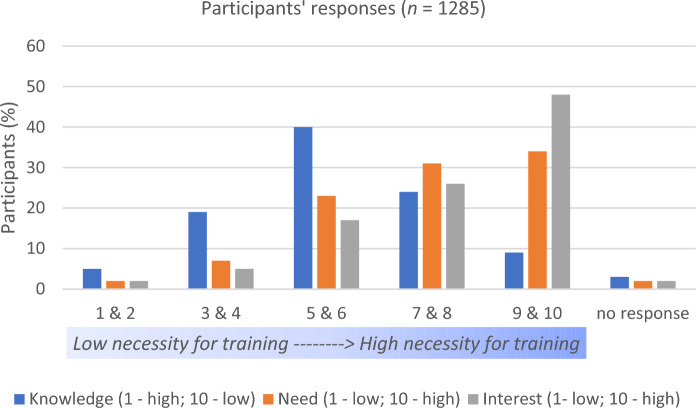
Necessity of staff training on hearing and communication. Staff perspective on necessity of training as indicated by pre-existing knowledge on the topic as well as need for and interest in a training program.

In the pre-training survey, most participants (40%) rated their knowledge level in regard to hearing and communication in their profession on a medium range between 5 and 6 on a scale from 1 to 10 (1 = very high knowledge; 10 = no knowledge) (Fig. 2). For the participants’ needs and interest in a training regarding hearing and communication in LTC facilities, the responses are distributed in ascending order from levels 1 and 2 (no need/interest) to 9 and 10 (very high need/interest). About a third of the sample (34%) indicated a very high need for training, and nearly half of the participants (48%) stated a very high interest with a level 9 and 10 (Fig. 2).

#### Post-training staff survey

**Fig. 3:**
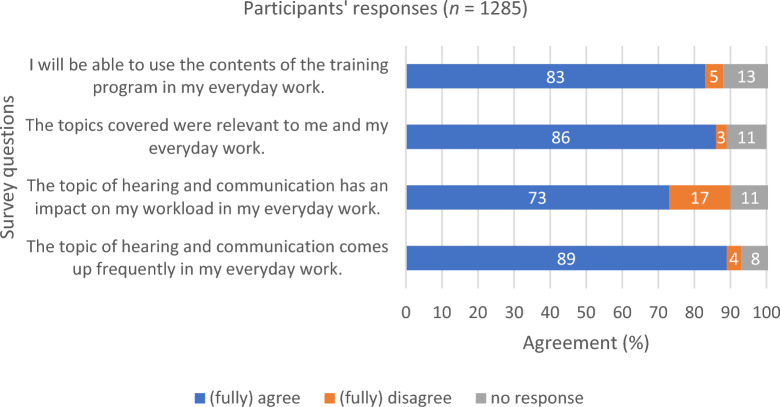
Participants’ agreement with statements about the preventive training program.

In the post-training survey, participants were asked about the perceived relevance of the training for their work and care routines, as well as their satisfaction with the training program (Fig. 3). 59% of participants fully agreed and 30% somewhat agreed with the statement ‘The topic of hearing and communication comes up frequently in my everyday work.’ Almost three quarters of respondents fully agreed (36%) or somewhat (37%) that the topic of hearing and communication in LTC facilities has an impact on their workload. When it came to an overall assessment of the training course, over half of the participants (58%) fully agreed and nearly a third (28%) somewhat agreed with the statement ‘The topics covered were relevant to me and my everyday work.’

The post-training survey also included questions about the participants’ knowledge and skills regarding hearing loss and hearing devices. After the training, 38% of participants fully agreed and 40% somewhat agreed with the statement ‘I am aware of the communicative effects of hearing loss.’ The ranking of the statement ‘I am aware of the emotional and social effects of hearing loss’ showed similar results. In addition to the effects of hearing loss, the training program also covered basic information about the use of hearing devices. Of the 1,285 participants, 20% fully agreed and 34% agreed somewhat with the statement ‘I can use hearing devices appropriately’ after the training course. In contrast, 16% somewhat disagreed and 6% fully disagreed. In the end, the majority of the participants (63%) felt overall well informed as a result of the training course.

### Acoustic and environmental accessibility

Results from the acoustic accessibility assessment indicate that most of the LTC facilities (81%) did not have specific concepts or measures regarding hearing accessible acoustics, e.g., sound-absorbing ceiling tiles or wall panels. One third of the facilities (29%) declared that they share information through the two-senses principle. This accessibility feature ensures the availability of any information via at least two senses. For example, information is given in a written (visual) and a spoken format (acoustic). However, the majority of facilities (60%) were not aware of this principle or did not use it when sharing information.

With regard to the reverberation time measurements, a substantial proportion of the assessed rooms in the LTC facilities exceeded recommended thresholds, particularly dining halls and multi-purpose rooms. The measurement had to be fully in the recommended scale of the DIN standard to pass. Even though in several cases the measurement or parts of the measurements were very close to the recommended scale, only 1% of the 277 rooms fulfilled the DIN standard.

**Fig. 4:**
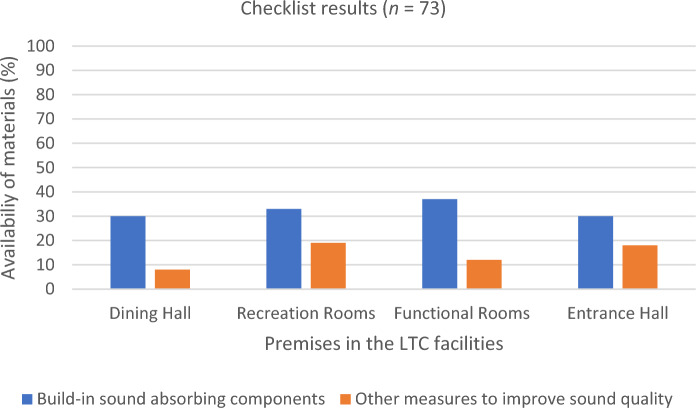
Availability of sound-absorbing components and other measures to improve sound quality in the LTC facilities.

Checklist data of the acoustic and environmental accessibility assessment revealed that there was a limited use of sound-absorbing materials and measures (Fig. 4). Build-in sound-absorbing components such as sound-absorbing ceilings and wall panels were found in only about a third of the common areas in the LTC facilities. Even scarcer were other measures to improve sound quality, such as carpeted floors, felt pads, curtains, or tablecloths, which were found in only 8 to 18% of the inspected common areas. At the same time, staff documented a frequent presence of background noises, for example, radio, TV, and clattering of dishes in most LTC facilities.

Staff members of the LTC facilities were able to accompany the acoustic and environmental accessibility assessment. In *n* = 71 LTC facilities a total of 179 employees participated and completed a questionnaire before and after the assessment. On average three persons accompanied the preventive training team at the assessment (min. = 1; max. = 6). Most of the participants were female (70%; male: 27%, diverse: 2%; missing data: 2%). The majority of the participants somewhat disagreed (42%) or fully disagreed (12%) with the statement ‘In my everyday work, I have paid attention to the room acoustics of our LTC facility.’ However, 31% somewhat agreed to the statement, and only 6% fully agreed. Participants were also asked to rate whether they had a positive impression of the room acoustics in their facility. Here, too, the most common responses were “somewhat disagree” (35%) and “fully disagree” (22%). On the other hand, 28% somewhat agreed and 9% fully agreed. Actions to improve room acoustics were presented to the LTC facilities and employees where necessary during the assessment and the wrap-up meeting with the facility’s management. These included relatively inexpensive solutions such as curtains and felt glides but also construction-related modifications. The participants’ perspective, whether they considered it realistic to implement structural modifications to improve room acoustics within a year, was rather cautious. Of the 179 participants, 36% somewhat agreed, 22% somewhat disagreed, and 15% fully disagreed. Only 10% stated they considered it realistic.

### Staff’s experiences with the implementation of the programs’ recommendations

The project team used data derived from the interviews and the follow-up online survey to analyze staff’s experiences in regard to feasibility and sustainability of the preventive training program in their LTC facility.

#### Interview results

Based on the analysis of the interview data, the project team focused on three overarching themes: enhanced awareness and motivation, organizational and structural factors, and the ambivalent role of the hearing champions.

#### Enhanced awareness and motivation

The participants of the interviews reported that the staff in general was more conscious of the topic of hearing loss and its effect on everyday life of the residents. They also expressed an increased awareness toward communication strategies and hearing device usage, e.g., checking if devices are worn and working. Moreover, several participants described more awareness in regard to acoustic accessibility, e.g., they reduce background noises, such as radios or TV, or they change to rooms with better acoustics for specific group activities.

#### Organizational and structural factors

On an organizational and structural level, the participants reported that the LTC facilities have to consider various aspects to improve the situation. Some of the participants stated that the documentation practices in regard to hearing and communication assessment of the residents improved after the preventive training program. However, other employees also mentioned this entails a higher workload for them. In two of the seven surveyed LTC facilities, network building with ENT professionals was successful. Other participants criticized that it was difficult to build cooperation in rural areas because there are either no ENT professionals nearby or they are not willing to come to the LTC facility.

Participants also reported challenges at the staff level. They mentioned competing priorities of caretaking, shortage of personnel, and staff turnover, resulting in missing time resources and workflow integration. Moreover, limited commitment on the part of management made it difficult to implement changes. Although the awareness for acoustic accessibility had risen, the participants pointed out that construction-related improvement takes time and financial resources. In addition, security and hygiene standards have to be considered.

#### The ambivalent role of the hearing champions

After the preventive training program, the hearing champions were mainly responsible for further engaging the implementation of the recommended actions with colleagues and management of the LTC facility. The interviewed hearing champions described an ambivalent picture of their role. On the one hand, they reported positive outcomes in their LTC facility because of their engagement. For example, they supported residents to receive hearing loss treatment and hearing devices, which had enhanced the quality of life of the residents and also improved the everyday work of staff members:

‘We all benefit from this. […] Residents have better quality of life when they hear better. It also benefits the nursing staff because I think it makes their work easier when residents can understand well. […] So, I think it really improves the quality of life for everyone. Improving quality of life here and in our interactions with each other for everyone’ (VE5_TN2, Social Care and Hearing Champion).

On the other hand, the hearing champions reported to have limited time resources and an overburdening workload. New tasks were often added on top of existing responsibilities, and in several cases the participants reported insufficient institutional support:

‘Since I actually work a lot of long shifts, when I have a break, I can grab a hearing aid at lunchtime and clean and check it. What’s really missing is the time for paperwork so that you can say, “Okay, I’ll call the ENT specialists again or see if I can find an appointment with the acoustician somewhere or that I can perhaps raise awareness among the residents’ relatives a little more.” There’s not really enough time for that’ (VE4_TN1, Social Care and Hearing Champion).

#### Follow-up survey results

The analysis of the follow-up survey data showed similar results to the post-training survey. The majority of the participants from this part of the study worked in social care (43%), followed by nursing staff (26%) and quality management personnel (14%). The rest of the participants (17%) stated that they work in another role, for example, in a management position or as a therapist. Most of the respondents somewhat agreed (62%) or fully agreed (22%) that the staff members in their LTC facility could accurately assess the hearing and communication abilities of the residents, while 16% somewhat disagreed. Only 12% fully agreed, whereas 66% somewhat agreed and 21% somewhat disagreed that ‘The staff at my LTC facility can respond appropriately to the hearing and communication abilities of the residents.’.

**Fig. 5:**
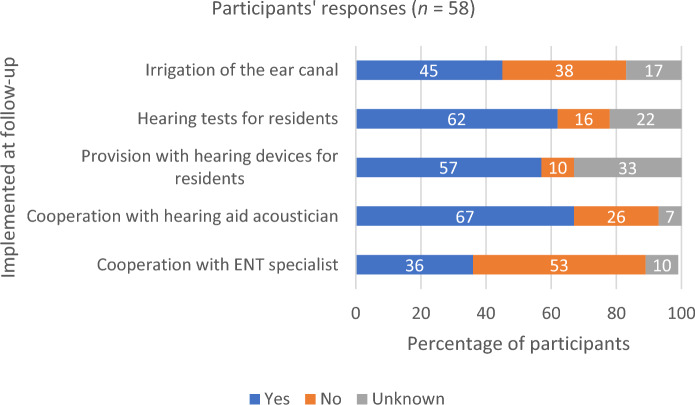
Implemented measures in the LTC facilities at 1-year follow-up after the preventive training program.

In the survey, 62% of the participants stated that after the preventive training program, hearing tests were carried out on more residents in their LTC facility (Fig. 5). In 16% of cases, there had been no further tests. Reasons for not carrying out further tests included that there was no perceived need, no cooperation with an ENT practice, and a lack of time. 45% of participants reported that residents regularly received irrigation of the ear canal if needed; however 38% did not report regular irrigations. More than half of the sample (57%) reported that residents in their LTC facility were provided with hearing devices after the training program. The survey results showed that the cooperation with ENT specialists and hearing aid acousticians was implemented to varying degrees of success. Over half of the participants responded that there was no regular cooperation with an ENT specialist (Fig. 5). 36% of participants reported that the LTC facility successfully built a cooperation after the preventive training program. Cooperation with an acoustician was implemented much more frequently (Fig. 5). In 67% of cases, respondents stated that cooperation had been in place since the preventive training program. Approximately a quarter of participants reported that there was no regular cooperation.

The majority of participants (86%) stated that no construction-related changes had been made in their LTC facility following the preventive training program. In infrequent cases, suspended ceilings were installed and new flooring was laid in some rooms. Approximately a quarter of participants reported changes to the interior design in the facility. Sound-absorbing elements such as acoustic pictures and panels or textiles such as curtains, placemats, and tablecloths were purchased after the program. According to the participants, (further) improvements would require (more) financial, time, and human resources as well as additional expertise. In some cases, the reason for not taking further measures was that a new building was being planned – improving the old building was therefore deemed unnecessary.

Approximately two-thirds of respondents (*n* = 34; 67%) were recruited as hearing champions for their LTC facility. The majority (85%) were still active in this role at the time of the online follow-up survey. Five people stated that they were no longer active as hearing champions. Reasons for this included a change of position within the facility, taking parental leave, and a lack of time due to other management responsibilities. In three cases, a successor had been appointed to take over the role. The majority of active hearing champions somewhat agreed (38%) or fully agreed (24%) that they were able to integrate their duties well into their everyday work. However, 35% of hearing champions were unable to integrate their duties well into their everyday work. Only in 8 out of 34 cases (24%), the LTC facility provided specific time resources for the hearing champions. It is notable that most of the participants (44%) did not provide any information on this. The majority of participants (59%) somewhat agreed, and 14% of respondents fully agreed that they have noticed overall sustainable changes in their institution as a result of the preventive training program. However, 17% somewhat disagreed and 2% fully disagreed with having noticed sustainable changes.

## Discussion

The study highlights critical gaps in acoustic accessibility and staff knowledge in LTC settings. Although the preventive program increased awareness and competence of staff regarding hearing and communication, structural and organizational barriers largely constrained sustained implementation.

### High interest in staff training on hearing and communication

The surveys on staff training showed that employees in LTC facilities are interested in more information on the topics of hearing and communication in older people, the care process, and how to use hearing devices. Staff of LTC facilities rated their previous level of knowledge on those topics as correspondingly low. This result is comparable to statements made by employees of care facilities in other studies^24,38,39^. In the present study, the majority was satisfied or even very satisfied with the content of the staff training. This suggests that a preventive training program regarding basic information about ear and hearing health, communication strategies for hearing loss, and handling and provision of hearing devices is a helpful and accepted method to meet LTC facility staff’s needs for training on the topic of hearing and communication.

### Improvements in hearing care in the LTC facilities

Feedback from the interviews and follow-up survey after the training program showed that participants applied communication strategies when supporting residents with hearing loss and gained more confidence in handling hearing aids. This relates to an improvement of hearing device provision for the residents: In order to encourage the use of hearing devices in individuals with hearing loss, regular checks of the devices, education, and support are necessary. Furthermore, the care environment must be made aware of the necessity in order to maintain the care processes and handle the hearing devices appropriately^11,16,40^. To increase the acceptance of hearing devices, it is also advisable to provide them as early as possible^1,13^, which is more likely to be facilitated by staff that is trained on the topic.

### Importance of monitoring hearing health

In terms of prevention for cognitive decline and dementia, monitoring hearing health and providing hearing devices at an early stage are important resources. Hearing loss is one of the most significant potentially modifiable risk factors for dementia^41^. LTC facilities have no influence on the hearing health of residents before they move in. However, when admitting new residents, they can assess existing hearing loss and difficulties and, if necessary, point out the possibilities of hearing tests and hearing device provision. The preventive training program has prompted several LTC facilities to expand their documentation to include this aspect of health care.

### Benefits and challenges of “hearing champions”

Feedback from the interviews and follow-up online survey indicates an ambivalent attitude towards the development of the role of hearing champions. On the one hand, dedicated hearing champions play a key role in the implementation and pursuit of improvements in LTC facilities. Once the role has been established in the facility, the hearing champions act as knowledgeable contact persons for colleagues, for residents, and for their relatives. They play a key role for the sustainable and long-term implementation of measures. On the other hand, due to limited time resources alongside their main duties, it was difficult for some employees to engage in this role. Particularly in facilities where only one person takes on the role, it was difficult to implement the measures and monitor the care processes. In some cases, tasks were performed on a voluntary basis outside of working hours. However, even hearing champions who work in a team reported challenges with time and organizational and structural challenges. These findings match with results from McShea and Ferguson^42^. In their study, they evaluated a training program for support workers to become “Hearing Champions”.

### Lack of acoustic accessibility

Results of the present study distinctly show that acoustic accessibility receives little to no attention in most LTC facilities. This is not only a regional problem, as studies from different countries show similar challenges^19,43^. There is no doubt that the fundamental problem for the facilities lies in the existing architecture and spatial layout. These are difficult to influence unless a new building or renovation is planned. In the present study, some facilities reported that the proposed measures were incorporated into the construction concept for new buildings. Typically, even in new buildings, acoustic accessibility is not necessarily taken into account in the planning. The incorporation seems to be a positive effect from the training program. It is important for both residents and staff to live and work in an acoustically pleasant environment. The assessment and improvement of acoustic conditions in LTC facilities are therefore essential^16,20,21,31,38^.

### Implications and future work

The overall results after the implementation of the preventive training program show that staff training may be an important step towards better hearing healthcare and improved acoustic accessibility in LTC facilities. However, for sustainable change and in accordance with the WHO’s H.E.A.R.I.N.G. package (Hearing Screening and Intervention, Ear Disease Prevention and Management, Access to Technologies, Rehabilitation Services, Improved Communication, Noise Reduction, and Greater Community Engagement)^5^, various stakeholders on different levels need to work together.

### Limitations

The evaluation of a preventive training program on hearing and communication for staff in LTC facilities gives valuable insights into the current practices and challenges of managing hearing loss in LTC. It was not possible to verify whether the improvements proposed in the preventive training program were subsequently implemented across the board because of participants and facilities that were lost to follow-up. The follow-up survey and interviews may have been biased by very dedicated participants who wanted to share the positive development in their facilities. However, participants did not only share progress but also discussed challenges in the interviews. During the implementation of the preventive training program and the evaluation, there were COVID-19 restrictions that limited data collection so that some facilities did not take part in all parts of the program. As the study was limited to one federal state in Germany (i.e., Bavaria), there may be restrictions to the generalizability of the results, and future studies are needed to evaluate the situation in other federal states.

### Implications for practice

The findings illustrate that hearing accessibility in LTC facilities must be viewed and addressed as a cross-sectional issue on multiple levels. On the *individual level* the residents themselves play an important role. Their motivation and attitudes towards hearing health as well as possible comorbidities should be taken into consideration. On an *interpersonal level* the LTC staff and relatives of the residents can influence the hearing health and hearing device provision of residents as well as the everyday communication with them. Specific training may be beneficial for those who lack the sufficient knowledge and skills required to support the residents. The organizational structures of LTC facilities also have an impact on hearing health and accessibility. At this *institutional level*, measures such as keeping records relating to ‘hearing and communication’, providing training for staff, and implementing measures to improve the acoustic accessibility of rooms can improve the situation. At a broader *structural level*, the focus is on the healthcare landscape and funding options. In this context, cooperation with ENT professionals plays a crucial role. Furthermore, contact with self-help groups could be expanded to obtain first-hand information on hearing loss and hearing devices. Insurance funds could use their existing resources to disseminate information on hearing health and accessibility to their policyholders, thereby raising awareness of these issues. LTC facilities rely on adequate financial and staffing resources to ensure holistic, people-centred care for their residents. To this end, the importance of improvement of hearing health care in LTC facilities must also be addressed at a political level.

## Conclusion

The results of the present study contribute to taking stock of the hearing health of older people with age-related hearing loss in Germany. This topic needs more attention, particularly among older people living in LTC facilities. The study revealed the necessity for various measures in LTC facilities to accommodate the residents’ hearing and communication needs. Improving hearing accessibility in LTC requires a dual strategy: enhancing staff competencies and optimizing acoustic environments. While the evaluated training interventions achieved meaningful improvements, structural and organizational limitations remained significant barriers. Sustainable change depends on institutional commitment and networked professional support, as well as systematic documentation and implementation into the workflow.

## Data Availability

The datasets generated during and/or analysed during the current study are available from the corresponding author on reasonable request.
